# TRPV6 Regulation by Cis-22a and Cholesterol

**DOI:** 10.3390/biom12060804

**Published:** 2022-06-08

**Authors:** Christina Humer, Sonja Lindinger, Aline L. Carrel, Christoph Romanin, Carmen Höglinger

**Affiliations:** 1 Institute of Biophysics, Johannes Kepler University of Linz, 4020 Linz, Austria; christina.humer_1@jku.at (C.H.); sonja_lindinger@gmx.at (S.L.); christoph.romanin@jku.at (C.R.); 2 Department of Chemistry and Biochemistry, University of Berne, 3012 Bern, Switzerland; aline.carrel@dcb.unibe.ch

**Keywords:** cholesterol, lipid, TRPV6, maximum current, slow calcium dependent inactivation (SCDI), LBS-2, PIP_2_

## Abstract

The highly calcium-selective transient receptor potential vanilloid-type channel TRPV6 is important for epithelial Ca^2+^ transport. Proper regulation of the inherently constitutively active TRPV6 channels is intricate in preserving Ca^2+^ homeostasis, whereby structural and functional data suggest that lipids hold an essential role. Altered expression levels or specific TRPV6 mutations may lead to diseases, hence, TRPV6 represents an interesting target for pharmacological modulation. Recent cryo-EM data identified that the specific TRPV6 blocker cis-22a binds, apart from the pore, to a site within the tetrameric channel that largely matches a lipid binding pocket, LBS-2. Therein, cis-22a may replace a lipid such as cholesterol that is bound in the open state. Based on site-directed mutagenesis and functional recordings, we identified and characterized a series of residues within LBS-2 that are essential for TRPV6 inhibition by cis-22a. Additionally, we investigated the modulatory potential of diverse cholesterol depletion efforts on TRPV6 activity. While LBS-2 mutants exhibited altered maximum currents, slow Ca^2+^-dependent inactivation (SCDI) as well as less inhibition by cis-22a, TRPV6 activity was resistant to cholesterol depletion. Hence, lipids other than cholesterol may predominate TRPV6 regulation when the channel is expressed in HEK293 cells.

## 1. Introduction

Calcium (Ca^2+^) is an essential intracellular messenger, competent in triggering versatile cellular responses such as secretion, gene expression, proliferation or cell death if the concentration of free cytosolic Ca^2+^ increases. Ca^2+^-based signal transduction and homeostasis rely on diverse, eventually cell-type or condition-dependently expressed proteins, including the transient receptor potential vanilloid-type channels TRPV5 and TRPV6 [1,2,3]. TRPV6 and closely related TRPV5 channels stand out among the member of the TRP superfamily of cation channels not only due to their high Ca^2+^ selectivity, but also in view of their inward rectifying current-voltage relationship and their constitutive activity [4,5,6,7,8]. Moreover, both channels are negatively modulated by entrant Ca^2+^ ions, which induce fast and slow Ca^2+^ dependent inactivation, FCDI and SCDI, respectively [7,9]. In addition, TRPV5 and TRPV6 channels serve as important components in epithelial Ca^2+^ transport, although they show deviations in the precise expression patterns. While TRPV5 is predominantly expressed in kidney, TRPV6 is more abundant in the duodenum [10]. Apart from mediating dietary Ca^2+^ uptake in these settings, TRPV6 is expressed in the pancreatic tissue, salivary gland, liver, esophagus, kidney, bone, testis and placenta (for review, see [11]).

Consistent with its importance in various tissues and cell types, there is an apparent link between TRPV6 mutations and several pathologies, including transient neonatal hyperparathyroidism (TNHP) and chronic pancreatitis (CP) [12,13,14]. In particular, nonsense or missense mutations that hinder Ca^2+^ uptake were reported to be overrepresented in patients suffering from CP compared to controls or to give rise to TNHP due to an insufficient Ca^2+^ transport through the placenta [12,14,15]. Moreover, changes in the expression level of TRPV6 have been identified in several types of cancer, with elevations representing a marker for poor prognosis in the case of breast cancer [16]. While also well established to be overexpressed in pancreatic cancer specimen, TRPV6 has instead been reported to be downregulated in esophageal squamous cell carcinoma and in renal cell carcinoma [16,17,18,19,20].

Altogether, TRPV6 channels represent an interesting therapeutic target, which may be addressed in different ways. A recent study demonstrated, for instance, that knockdown of TRPV6 via RNA interference reduces the invasiveness of LNCaP prostate cancer cells, their migration abilities, wound healing and proliferation [19]. Conditions of elevated TRPV6 expression levels or an increased activity in the channel may also be targeted with inhibitors of the channel itself rather than interfering with protein synthesis. Among them rank the rather unspecific 2-aminoethyl diphenylborinate (2-APB) and ruthenium red (RR) as a universal ion channel blocker, while the antifungal drug econazole represents a more specific modulator of TRPV6 [21,22,23]. Additionally, Soricidin, a peptide present in the paralytic venom of the northern short-tailed shrew, and specific C-terminal segments thereof (SOR-C13 and SOR-C27), were reported to have an antagonistic effect on TRPV6, whereof SOR-C13 has even reached clinical trials [24,25,26]. Moreover, compounds containing a phenyl-cyclohexyl-piperazine scaffold were proven to preferentially inhibit TRPV6 rather than related TRPV5 channels, which might be further supplemented with a photo-switchable moiety for spatiotemporal manipulation [27,28]. One of the most potent variants is in this regard cis-22a, which is characterized by a half-maximum inhibitory concentration (IC_50_) of 82 ± 25 nM, as well as 3OG, representing an analogue with almost the same potency, which is modified with structural elements of capsaicin [27,29,30].

TRPV6 channels are formed by the assembly of four TRPV6 proteins in a symmetrical tetramer, whereof each subunit contains six transmembrane domains (S1 S6) with a short pore loop intercalated between S5 and S6, as well as a cytosolic N-and C-terminus. The S1–S4 domain and the pore domain, the latter of which is formed by the S5 helix, the pore loop and S6, engage in a swapped configuration. Thereby, the pore domain of an individual subunit interacts with the S1–S4 domain of an adjacent subunit rather than the one of the same protein [31,32]. As initially revealed in cryo-EM data on human TRPV6 of McGoldrick and colleagues, the overall channel assembly harbors 16 regions, 4 per TRPV6 subunit, with densities likely represented by lipids rather than originating from the proteins themselves [32]. Accordingly, these densities are referred to as lipid binding sites 1 4 (LBS1 4). Interestingly, the size of lipid binding site 2 (LBS-2) has been reported to change in dependence of the open or closed state of the channel. This is consistent with the common tenets that lipids play an important role in TRPV6 activity [32].

The surfaces of the lipid binding sites are formed by the side chains and carbonyl oxygens of residues of the transmembrane domains but also involve moieties of the cytosolic portions of the protein. LBS-2, for instance, maps to the cytosolic half of the membrane where it is interlaced between the third and fourth transmembrane domain as well as the S4 S5 linker of one subunit and the pair of transmembrane helices of the pore domain of an adjacent subunit. Additionally, the TRP helix that follows S6 and aligns parallel to the membrane due to its amphipathic character is appropriately placed to interact with lipids [11,32]. According to McGoldrick et al., the chemical properties of the residues lining density four (LBS-4) are predestined to coordinate phospholipids with positively charged head groups and the density would indeed fit phosphatidylcholine or phosphatidylethanolamine. In contrast to LBS-4, the nature of the presumptively associated lipids is not that conclusive regarding the other three densities. Structurally, the LBS-2 density would principally fit cholesterol or cholesterol hemisuccinate (CHS). Thereby, awareness is needed that the buffers implemented upon purifying hTRPV6 contained rather high concentrations of CHS. Thus, it seems possible that those lipids bound under natural conditions has been shown to be, at least in parts, replaced in the excess of CHS [32]. Yet, consistent with possible binding sites of cholesterol within the channel, there are also functional data indicating that in Jurkat T cells, TRPV6 activity is sensitive to the depletion of this specific lipid [33,34]. Apart from cholesterol, different functional approaches point to phosphatidylinositol-4,5-bisphosphate (PIP_2_) as an important regulator of TRPV6 and that specific channel characteristics are dependent on the levels of this lipid [35,36,37,38,39,40,41]. Moreover, for other members of the TRP superfamily, including polycystin-type channels, TRPM4 and TRPM8 as representatives of the melastatin-type subfamily or the vanilloid-type channels TRPV1 and TRPV5, there is large experimental evidence for their regulation by PIP_2_. In some instances, the most likely binding sites have been resolved structurally [42,43,44,45,46,47]. Considering cryo-EM data of human TRPV6, however, none of the presumptive lipid densities matches the shape of PIP_2_ properly [32].

Interestingly, cryo-EM data on cis-22a-complexed TRPV6 strongly supports that one binding site of the inhibitor overlaps with LBS-2, whereby occupancy with cis-22a seems to go along with the replacement of the lipid. Yet, apart from binding to membrane-embedded regions, another binding site of the blocker has been identified: the pore itself [30]. Comparison of the inhibitor-bound TRPV6 structures of the open, closed and inactivated state suggests that binding of cis-22a and related substances to the open pore of the channel serves as a mimetic of calmodulin binding, the latter of which causes inactivation under native conditions. The α-to-π helical transition occurring within S6 of the open rather than the closed channel remains intact upon cis-22a engagement. Yet, as a consequence of attraction by the bound inhibitor, lower sections of the respective helix are displaced towards the center of the pore. The resulting constriction within the gate region was proposed to give rise to a hydrophobic seal that prevents ion permeation [30].

So far, the main focus has been led on the pore binding site, as this location apparently serves as the main binding site for the inhibitor. In the present study, however we investigated the association of cis-22a with LBS-2 using site-directed mutagenesis, calcium imaging and patch clamp recordings. Thereby, we identified several TRPV6 mutants: Q483K, K484A, G488R and Q596E, which reveal significant reductions in the inhibition by 0.1 μM and 10 μM cis-22a upon expression in HEK293 cells. Moreover, characteristics of the mutant channels such as the maximum current supported and SCDI are significantly different to TRPV6 WT. Since the mutations drastically alter the properties of residues which directly line LBS-2 and given that Q483 and K484 have already been implicated in lipid recognition [32,37], we further addressed the controverse of whether the lipid that binds to LBS-2 was cholesterol, also upon expression in HEK293 cells, or if the structural match was an artifact. Therefore, we took advantage of three different approaches to interfere with potential TRPV6-cholesterol interactions: treatment with methyl-β-cyclodextrin (MβCD), application of filipin and exposure to cholesterol oxidase (CO). Yet, our data disfavor a regulatory role of cholesterol on TRPV6 overexpressed in HEK cells, given that none of the different strategies had a significant effect on TRPV6 maximum current and SCDI or, as tested for CO, on the response to cis-22a.

## 2. Materials and Methods

### 2.1. DNA Constructs and Reagents

Human TRPV6 (hTRPV6) was present in the pTagRFP-C vector, which was kindly provided by R. Bhardwaj and M. Hediger (University of Bern, Switzerland). The 725 aa long variant of hTRPV6 that lacks the N-terminal most in the 40 amino acids of the in vivo expressed protein (accession number NM_018646.6) was deployed throughout all experiments. pTagRFP-TRPV6 mutants (Q483K, K484A, G488R and Q596E) were generated using the QuickChange XL site-directed mutagenesis kit (Stratagene, CA, USA). Forward and reverse mutagenesis primers were synthesized by Eurofins genomics GmbH (Vienna, Austria) and correctness of the constructs was confirmed by sequencing (Microsynth Austria GmbH, Vienna, Austria). Human Orai1 (Orai1, accession number NM_032790.3), N-terminally cherry-myc-tagged (vector backbone pEX-GWI), was kindly provided by Ricardo Dolmetsch’s lab (Stanford University, Stanford, CA, USA). Human STIM1 (STIM1, accession number NM_003156), which was labeled at the N-terminus with ECFP (pECFP-C1 vector) was kindly provided by T. Meyer’s lab (Stanford University). The TRPV6 inhibitor, cis-22a, was synthesized and provided by the lab of Jean-Louis Reymond (University of Bern, Switzerland).

### 2.2. Cell Culture and Transfection

Human embryonic kidney (HEK) 293 cells were cultured in DMEM supplemented with 10% fetal calf serum, penicillin (100 U/mL) and streptomycin (100 µg/mL) at 37 °C under humidified atmosphere containing 5% CO_2_. Transient transfection was performed 20–24 h before Fura-2 or electrophysiological experiments using the TransFectin Lipid reagent (Bio-Rad, Hercules, CA, USA), according to the instructions of the manufacturer. To exclude mycoplasma, contaminations, tests were performed on a regular basis using the VenorGem Advanced Mycoplasma Detection kit (VenorGEM).

### 2.3. Calcium Imaging

For Fura-2 experiments, HEK293 cells were seeded on poly-lysine coated cover glasses 24 h prior to transfection. In preparation of the measurements, the coverslips were washed thoroughly with nominally Ca^2+^-free extracellular solution (0 mM Ca^2+^ ECS; 140 mM NaCl, 5 mM KCl, 1 mM MgCl_2_, 10 mM HEPES, 10 mM glucose, pH adjusted to 7.4 with NaOH) to remove medium remnants. Followingly, cells were loaded with 1 μM Fura-2-AM (SIGMA Life Science, 47989-1MG-F) in 0 mM Ca^2+^ ECS for 25 min in a dark state at room temperature. Fura-2 was dissolved in dimethyl sulfoxide (DMSO; Merck, Darmstadt, Germany) in stocks of a concentration of 1 mM without further additions, respectively. After removing the dye-loading solution and a further washing step with 0 mM Ca^2+^ ECS, incubation was continued for another span of 25 min to promote complete de-esterification of the dye. Subsequently, glass slides were mounted on an Axiovert 135 inverted microscope (Zeiss, Germany), supplemented with a sCMOS-Panda digitale Scientific Grade camera 4.2 MPixel Rolling Shutter Version (14401303) and a LedHUB LED Light-Engine (2200430) as light source (LedHUB®; Omicron-Laserage Laserprodukte GmbH, Rodgau, Germany) light source, which was controlled using Omicron Control Center v3.9.28. A CHROMA 69008× ET ECFP/EYFP/mCherry 25mm Dia Mounted filter was used for identifying cells expressing TagRFP-labeled TRPV6 proteins and a FURA dichroic mirror (OMEGA OPTICAL XF114, Omega Optical Holdings, LLC Brattleboro, USA) for the actual calcium imaging experiment, which was controlled with the VisiView software 4.5.0 (Visitron Systems GmbH, Puchheim, Germany). During the measurement, cells were initially consecutively exposed to Ca^2+^-free ECS and 2 mM Ca^2+^-containing ECS (140 mM NaCl, 5 mM KCl, 1 mM MgCl_2_, 2 mM CaCl_2_, 10 mM glucose and 10 mM HEPES buffer; pH adjusted to pH 7.4 with NaOH), 1 min each. After perfusion with 0 mM Ca^2+^ ECS for 2 min, 2 mM Ca^2+^ ECS was re-applied for a further minute. To investigate the effect of cis-22a on internal Ca^2+^ levels, perfusion continued with 2 mM Ca^2+^ + DMSO (0.1 vol%) for 1 min as internal solvent control and then with 0.1 or 10 µM cis-22a in 2 mM Ca^2+^ ECS, 2 min each. To altogether prevent Ca^2+^ influx, cells were finally exposed to Ca^2+^ free solution for 2 min. Changes in intracellular Ca^2+^ were monitored for single cells upon alternatingly exciting Fura-2 with 340 nm and 385 nm with an exposure time of 100 ms and an interval between successive iterations of 10 s. Emission was monitored at a constant wavelength of 505 nm for each excitation wavelength, respectively. For ratiometric measurements, the ratio of the emission intensities detected upon excitation at 340 nm and 385 nm was determined after background subtraction. All mutants were measured on at least two days at room temperature.

Statistical analysis and blotting relied on the OriginPro 2020b software. F_340nm_/F_385nm_ curves recorded for each cell were further normalized to the respective minimal value for a better comparability. After this normalization step, the fold-changes in cytosolic Ca^2+^ at the time point t = 250 s were extracted as a read-out of the maximal channel activity, the percentage decrease in the normalized F_340nm_/F_385nm_ from t = 250 s to t = 350 s served as an indication for alterations in channel inactivation and from t = 350 s when inactivation reached steady-state to t = 580 s for analyzing inhibition by 10 μM cis-22a, respectively. Data were tested for normal distribution and the presence of possible outliers, whereafter statistical significance between TRPV6 WT and the individual mutants was evaluated using either unpaired two-sided Student´s t-tests for normally distributed data or Mann–Whitney U tests if a normal distribution had to be rejected. Within bar graphs (mean ± SEM), statistical significance (*p* < 0.05) is indicated by `*´.

### 2.4. Electrophysiology

For patch clamp measurements, cells were harvested 20 h after transfection and re-seeded on poly-lysine coated cover slips. After incubation at 37 °C for about 4 h for recovery and attachment, electrophysiological experiments were performed at RT (20 °C to 24 °C), using the patch-clamp technique in the whole-cell configurations. An Ag/AgCl electrode was used as a reference electrode. Voltage ramps were applied every 5 s from a holding potential of 50 mV in the case of TRPV6 measurements, covering a range of −90 to +90 mV over a time period of 200 ms. For CRAC currents, the holding potential was set to 0 mV and the duration of the voltage ramp was 1 s, respectively. For the time courses, the inward current density at −74 mV was extracted and plotted over time. The internal pipette solution contained 145 mM cesium methanesulfonate, 8 mM NaCl, 3.5 mM MgCl_2_, 10 mM Hepes, and 20 mM EGTA (pH 7.2). The standard extracellular solution consisted of 145 mM NaCl, 5 mM CsCl, 1 mM MgCl_2_, 10 mM Hepes, 10 mM glucose, and 10 mM CaCl_2_ (pH 7.4). All currents were leak-corrected by subtracting either the remaining currents after 1 mM La^3+^ application at the end of the experiment (for TRPV6 currents) or the initial voltage ramps obtained shortly after break-in with no visible current activation (for CRAC currents). The liquid junction potential was determined as 12 mV; the applied voltages were not adjusted. All experiments were conducted on a minimum of two different days.

Statistical analyses were carried out and graphs (means ± SEM) were created using the OriginPro software. Data were tested for normal distribution and statistical significance between TRPV6 WT and the individual mutants was evaluated using either unpaired two-sided Student´s t-tests for normally distributed data or Mann–Whitney U tests if normal distribution was rejected. `*´ indicates significance (*p* value < 0.05).

## 3. Results

### 3.1. Structure-Guided Mutagenesis within LBS-2 of Human TRPV6

Cryo-EM structures of TRPV6 showed that the size of LBS-2 decreases in the closed state compared to the open channel, what might be interpreted as a major role of LBS-2 in channel regulation. We therefore took advantage of the available structural data and mutated several residues within LBS-2 that were supposed as key determinants of the chemical environment for this site or to directly coordinate the bound lipid.

According to McGoldrick et al., the chemical milieu within LBS-2 is, due to the residues R470, Q483, K484 and Q596, suitable for charged-based and polar interactions with cholesterol or with these phospholipids containing negatively charged head groups. Moreover, comparison of the open and presumptively closed TRPV6 channel suggests that Q483 interacts with the bound lipid in the open state, while structural alterations observed in the closed state would cause clashing of the respective side chain with the lipid if the density remained unchanged. Ca^2+^ uptake of TRPV6 Q483A has been reported to be significantly slower compared to that of TRPV6 WT, further strengthening the relevance of this residue within LBS-2. In the present analysis, we focused on the effect of strengthening a potential interaction of the bound lipid with this site by replacing the glutamine with a lysine residue (TRPV6 Q483K). Vice versa, an acidic amino acid (glutamate) was inserted instead of Q596 to potentially interfere with the binding of negatively charged lipids. Although it is a common practice to substitute positively charged residues with glutamine to lower presumptive interactions with negatively charged lipids (e.g. conducted in [37]), we herein replaced naturally present basic residues with alanine to potentially weaken LBS-2 given the potential role of inherently present glutamine residues in forming LBS-2. Although a reduced channel activity in TRPV6 K484Q has already been published by Cai et al. [37], we replaced K484 by alanine (TRPV6 K484A) to completely abolish the assumed lipid interaction at this site. Another basic residue within LBS-2 is R470. Yet, in the present study, we refrained from presenting data on R470 mutants, as this specific position has already been closely investigated in previous studies [30,32,37]. Apart from structural insights and structure-guided mutagenesis, a publication by Velisetty et al. requires attention upon intending to investigate LBS-2. In the concerning study, sequence alignments highlighted the absence of a positively charged residue within the region connecting the fourth and fifth transmembrane domain of TRPV5/6 compared to TRPV1 (TRPV1 R575), presumptively contributing to differences in lipid recognition. In the case of TRPV6, the equivalent position is G488 [36]. Although Velisetty and colleagues have presented evidence that the G488R mutation increases the affinity of TRPV6 for PIP_2_ [36], this mutant was also included in the current study for a deeper investigation.

### 3.2. Point Mutations within LBS-2 Alter TRPV6 Activity and Cytosolic Ca^2+^ Levels in Fura-2 Measurements

**Figure 1:**
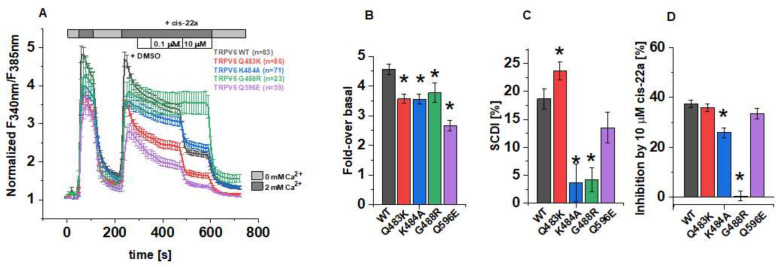
**Functional characterization of TRPV6 mutants in Fura-2 experiments.** (**A**) Changes in the intracellular Ca^2+^ level of HEK293 cells overexpressing human TRPV6 WT or the respective mutant upon perfusion with Ca^2+^-free extracellular solution (ECS), 2 mM Ca^2+^-containing ECS, 0.1 vol% DMSO, 0.1 µM and 10 µM cis-22a in 2 mM Ca^2+^-containing ECS, as indicated by the bars. Fura-2 emission was monitored in intervals of 10 s upon alternating excitation at 340 nm and 385 nm. After background subtraction, the ratio of the respective emission intensities was determined (F_340nm_/F_385nm_) and normalized to the minimum F_340nm_/F_385nm_ for each cell. Shown are averaged time courses (mean ± SEM) of 23–86 cells, measured on 2-4 days. (**B**) Block diagram summarizing fold-changes in the intracellular Ca^2+^ levels at the time point t=250 s according to measurements in (**A**). (**C**) Block diagram of the decrease in intracellular Ca^2+^ from t = 250 s to t = 350 s of the measurements in (**A**), serving as indicator for the slow Ca^2+^-dependent inactivation. (**D**) Block diagram of the inhibition by 10 µM cis-22a in % according to measurements in (**A**). The asterisk (*) indicates the statistical significance (*p* < 0.05) in comparison to TRPV6 WT.

The effects of the afore mentioned LBS-2 mutations on the activity of TRPV6 were initially investigated in Fura-2 calcium imaging experiments. Within a single measurement, cells were at first alternatingly perfused with 0 mM and 2 mM Ca^2+^ ECS, following the application of dimethyl sulfoxide (DMSO), cis-22a and finally, again 0 mM Ca^2+^ ECS. The recordings were conducted in a ratiometric regime, and these data were further normalized for each single cell to the minimum value for a better comparability (Figure 1A). Thereof, the fold-changes in F_340nm_/F_385nm_ over the basal (minimal) value at the time point t = 250 s serve to determine if the individual mutations alter the maximum activity in the respective channel. Indeed, all of the TRPV6 mutants (Q483K/K484A/G488R/Q596E) showed significantly lower maximum increases in the cytosolic Ca^2+^ level compared to the wild-type control if transiently overexpressed in human embryonic kidney (HEK) 293 cells (Figure 1B). Thereby, the TRPV6 Q596E mutation had the strongest effect, while the consequence of neutralizing or introducing positively charged residues as in the case of K484A, Q483K and G488R was moderate and rather similar in the extent of interfering with increases in cytosolic Ca^2+^.

One important biophysical characteristic of TRPV6 channels is their slow Ca^2+^-dependent inactivation (SCDI). Although the monitoring of changes in the intracellular Ca^2+^ level not only provides exclusive insights on the state of activity in plasma membrane channels but it is also impacted by the concurrent activity in, e.g., transport systems that remove Ca^2+^ from the cytosol, we tried to pre-investigate the inactivation behavior using Fura-2. For TRPV6 WT, the fold-change in cytosolic Ca^2+^ decreased from the time point t = 250 s to t = 350 s by about 19%. Interestingly, this decrease was significantly lower for TRPV6 K484A and TRPV6 G488R (Figure 1C), what would be consistent with a reduced SCDI for both mutants, assuming that the activity of endogenous Ca^2+^ transport and buffering systems remained unchanged. Consistently, also Velisetty et al. reported Ca^2+^-induced inactivation of TRPV6 to be eliminated after introducing the point mutation G488R [36]. Moreover, we experienced that the number of viable, transfected cells was very low in the case of TRPV6 G488R compared to those expressing TRPV6 WT or the other mutants while basal F_340nm_/F_385nm_ values were rather high for TRPV6 G488R expressing cells (Supplementary Figure S1). Both of the mentioned observations might indicate a severely compromised inactivation mechanism and represent the consequence of unhindered Ca^2+^ influx throughout cultivation in conventional cell culture medium. Although SCDI was also decreased for TRPV6 Q596E, this alteration was not significant compared to TRPV6 WT, while intracellular Ca^2+^ dropped rapidly between the above mentioned time points in the case of the TRPV6 Q483K.

It has become evident in cryo-EM data that apart from lipids, LBS-2 also serves as a binding site for the small molecule inhibitor cis-22a. [30]. Given this overlap, we attempted to investigate if the mutations in LBS-2 affect binding of cis-22a as well. Therefore, cells were exposed to two different concentrations of the inhibitor, 0.1 μM and 10 μM, respectively, after perfusing DMSO-supplemented (0.1 vol%) 2 mM Ca^2+^ ECS as an internal solvent control. While no effects of the solvent control on channel activity were discernible, we observed some response to the lower blocker concentration. However, the effect of 0.1 μM cis-22a on individual cells was rather variable (Supplementary Figure S1) so that we decided to focus on the higher blocker concentration (10 µM) in Fura-2 measurements. As summarized in Figure 1D, the deviation from the inhibition of approximately 37% (±1.4%) of TRPV6 WT was most pronounced for the glycine-to-arginine mutant. Indeed, TRPV6 G488R showed only a marginal residual mean inhibition of 0.4% (±2.0%). Vice versa, with a reduction in cytosolic Ca^2+^ by 35.8% (±1.5%), cis-22a displayed the strongest inhibitory potential on TRPV6 Q483K among all the mutants. The same mutation was already included in a previous paper on the inhibition of TRPV6 by cis-22a but was in this regard only analyzed in a cadmium-influx assay probing the response to 0.25 μM of the blocker [30]. While the response to this concentration was statistically significantly lower compared to TRPV6 WT, the presently determined inhibition by the high blocker concentration was within the range of the wild-type control, as is also valid for TRPV6 Q596E.

Collectively, pre-evaluations in Fura-2 experiments revealed differences in the fold-change of cytosolic Ca^2+^, its time-dependent decay and eventually, the response to 10 µM cis-22a of TRPV6 Q483K, K484A, G488R and Q596E mutants compared to the wild-type channel.

### 3.3. Mutations in LBS-2 Lead to Reduced Maximum Currents, SCDI and Inhibition by Cis-22a

**Figure 2:**
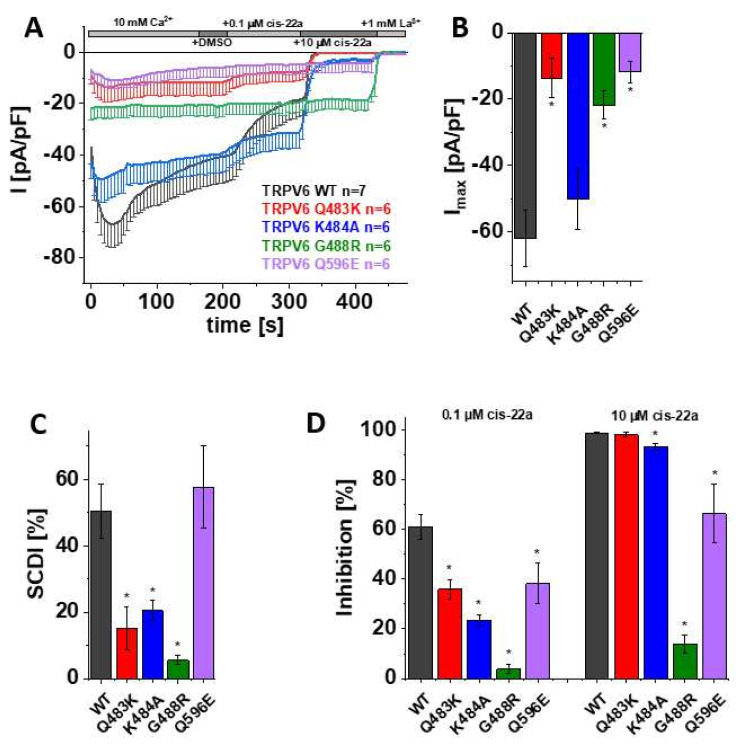
**Mutations in LBS-2 lead to reduced maximum currents, SCDI and inhibition by cis-22a.** (**A**) Averaged time course of whole-cell currents (mean ± SEM) recorded from HEK 293 cells expressing TRPV6 WT (black), TRPV6 Q483K (red), TRPV6 K484A (blue), TRPV6 G488R (green) and TRPV6 Q596E (purple). Throughout the experiment, application of 10 mM Ca^2+^ was followed by consecutive addition of DMSO, 0.1 µM cis-22a, 10 µM cis-22a, and 1 mM La^3+^, as indicated by the grey bars. (**B**) Block diagram of maximum currents according to measurements in (**A**). (**C**) Block diagram of the extent of SCDI in % according to measurements in (**A**). (**D**) Block diagram of the extent of inhibition in % by 0.1 µM (left) and 10 µM (right) cis-22a according to measurements in (**A**). The asterisk (*) indicates the statistical significance (*p* < 0.05) in comparison to TRPV6 WT.

As mentioned before, the information obtained in the calcium imaging experiments as presented herein includes changes in the cytosolic Ca^2+^ level. To directly monitor Ca^2+^ influx through the channel rather than the consequent alterations in the intracellular concentration of the ion, the distinct mutants and TRPV6 WT were further elucidated in electrophysiological recordings. Thereby, patch clamp measurements, which were conducted in the whole-cell mode throughout this study, revealed a significantly reduced maximum current of TRPV6 Q483K compared to TRPV6 WT (Figure 2A,B). This is consistent with the already mentioned lower fold-change in cytosolic Ca^2+^ extracted from Fura-2 recordings. Additionally, slow Ca^2+^-dependent inactivation of TRPV6 Q483K was significantly less pronounced compared to the wild-type channel, what also applies to TRPV6 K484A (Figure 2A,C). Although TRPV6 K484A also showed lower mean maximum currents compared to the wild-type control, rather high, inherent variations in the TRPV6 current size obviated these results to obtain statistically significant results (Figure 2B). TRPV6 G488R revealed no SCDI, which is in line with Velisetty et al. [36], and the maximum current was significantly decreased in comparison with TRPV6 WT as well (Figure 2A–C). While the G488R substitution is deemed to strengthen lipid binding, a weakening thereof by replacing a glutamine by an acidic residue (Q596E) had surprisingly the same consequence on maximum currents. However, SCDI of TRPV6 Q596E remained within the range of the wild-type control (Figure 2A–C).

After demonstrating that mutations within LBS-2 impose effects on maximum currents and SCDI, as well as given that Fura-2 experiments already indicated a lower sensitivity of some mutants to the inhibition by cis-22a, we investigated the inhibitory potential of cis-22a more closely in electrophysiological recordings. We again ensured the absence of solvent effects upon analyzing the response to the inhibitor by applying DMSO (1 µL/mL) to the extracellular solution via the perfusion system after TRPV6 currents reached a plateau. Subsequently, cells were exposed to 0.1 µM cis-22a, which approximately equals the IC_50_ of the inhibitor on TRPV6 WT. When the inhibition of the Ca^2+^ current by the lower concentration reached a steady-state, treatment was continued by applying 10 µM cis-22a, a concentration at which TRPV6 WT currents are normally blocked by around 98% in electrophysiological recordings, as established in [30]. Indeed, all of the mutants revealed significantly decreased inhibition by 0.1 µM cis-22a compared to TRPV6 WT (Figure 2A,D). Except Q483K, all mutants were also significantly less sensitive to the application of 10 µM cis-22a. This is in line with the previously described Fura-2 measurements, where TRPV6 Q483K preserved the highest extent of inhibition by cis-22a compared to the other mutants. Additionally, in line with calcium imaging experiments, TRPV6 G488R was rather resistant to 10 µM cis-22a in electrophysiological recordings, with inhibition reaching about 14% (Figure 2A,D).

Together, these data suggest that main features of the channel like maximum current, SCDI and inhibition by the small molecule blocker cis-22a are regulated by the integrity of LBS-2 and thereby presumably its lipid binding abilities. As mentioned in a previous section, cholesterol or CHS is likely bound in the cryo-EM structure of open TRPV6 (McGoldrick, 2018 no. 24). In a next step, we investigated the behavior of the wild-type channel with respect to the mentioned properties upon depletion of the lipid cholesterol by external treatments rather than mutational modification of the binding site. Thereby, an eventual impact of structural changes for the point mutations, which cannot be excluded so far, is circumvented so that these experiments are likely better suitable for investigating lipidic regulation under of the native channel.

### 3.4. Preincubation with Cholesterol Oxidase (CO) Has No Effect on Maximum Currents and SCDI of TRPV6 WT

**Figure 3:**
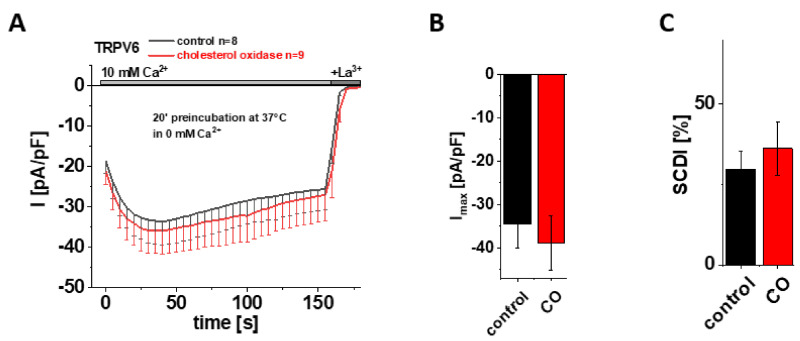
**Preincubation with cholesterol oxidase (CO) has no effect on maximum currents and SCDI of TRPV6 WT.** (**A**) Averaged time course of whole-cell currents (mean ± SEM) recorded from HEK 293 cells expressing TRPV6 WT. Cells were preincubated in 0 mM Ca^2+^ extracellular solution with (red) and without (control, black) 2 U/mL CO at 37 °C for 20 min. Measurements were carried out in 10 mM Ca^2+^ extracellular solution and currents were blocked by 1 mM La^3+^ at the end of the experiments, as indicated by the grey bars. (**B**) Block diagram of maximum currents according to measurements in (**A**). (**C**) Block diagram of the extent of SCDI in % according to measurements in (**A**). There is no statistical significance (*p* < 0.05) in comparison to TRPV6 WT.

Based on their cryo-EM data, McGoldrick and colleagues suggested cholesterol or CHS, which was abundant upon protein purification, as the most likely lipid captured within LBS-2 in the open state [32]. After having shown that site-directed mutagenesis within this binding site significantly reduces the maximum current and SCDI of TRPV6, it was tempting to hypothesize that these characteristics were affected upon depleting cholesterol from the membrane as well, supposing that it is cholesterol that mainly binds to LBS-2 also in a cellular environment. To test this hypothesis, we initially applied cholesterol oxidase (CO) as an approach for cholesterol depletion. Cholesterol oxidase catalyzes the conversion of cholesterol into cholest-5-en-3-one and a subsequent isomerization reaction into cholest-4-en-3-one, the latter of which functionally deviates from cholesterol [48]. Treatment with CO thereby reduces the amount of cholesterol within the membrane, with the consequence of a distortion in the liquid-ordered phase of the bilayer and interfering with cholesterol–protein interactions [48,49,50]. Hence, we preincubated HEK293 cells, transiently transfected with TRPV6 WT, in 0 mM Ca^2+^ solution with 2 U/mL CO at 37 °C for 20 min. For control experiments, cells were preincubated for 20 min at 37 °C in 0 mM Ca^2+^ solution without the addition of CO. Contrary to the above mentioned hypothesis, patch clamp measurements in the whole-cell mode revealed no significant difference between CO treated and control cells, neither for maximum currents nor for the extent of SCDI (Figure 3A–C). To ensure that the treatment itself was successful and to exclude conclusions drawn on the basis of experimental failure, additional control experiments were performed with HEK293 cells transiently transfected with Orai1 and STIM1. These measurements revealed significantly increased currents upon 20 min preincubation with CO in comparison to cells which were preincubated without CO (Supplementary Figure S2A,B), consistent with results of Derler et al. on the effect of cholesterol depletion on CRAC channel activity [51].

### 3.5. Application of MβCD Lacks a Significant Effect on Maximum Currents and SCDI of TRPV6 WT

**Figure 4:**
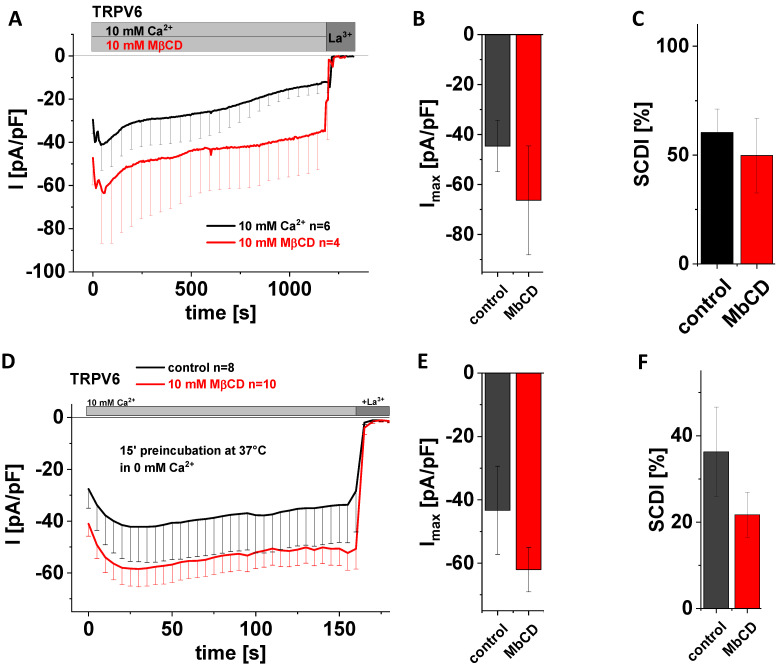
**Application of MβCD has no effect on maximum currents and SCDI of TRPV6 WT.** (**A**) Averaged time course of whole-cell currents (mean ± SEM) recorded from HEK293 cells expressing TRPV6 WT. Measurements were carried out in 10 mM Ca^2+^ extracellular solution with (red) or without (control, black) 10 mM MβCD and currents were blocked by 1 mM La^3+^ at the end of the experiments, as indicated by the grey bars. (**B**) Block diagram of maximum currents according to measurements in (**A**). (**C**) Block diagram of the extent of SCDI in % according to measurements in (**A**). (**D**) Averaged time course of whole-cell currents (mean ± SEM) recorded from HEK293 cells expressing TRPV6 WT. Cells were preincubated in 0 mM Ca^2+^ extracellular solution with (red) and without (control, black) 10 mM MβCD at 37 °C for 15 minutes. Measurements were carried out in 10 mM Ca^2+^ extracellular solution and currents were blocked by 1 mM La^3+^ at the end of the experiments, as indicated by the grey bars. (**E**) Block diagram of maximum currents according to measurements in (**D**). (**F**) Block diagram of the extent of SCDI in % according to measurements in (**D**). There is no statistical significance (*p* < 0.05) in comparison to TRPV6 WT.

A very prominent way to study protein–cholesterol interactions is to treat cells with methyl-beta-cyclodextrin (MβCD). The latter is a water-soluble oligosaccharide that forms dimers containing a hydrophobic cavity, wherein cholesterol gets captured and thereby extracted from the membrane [52,53]. The great advantage of this effort for interfering with the interaction between cholesterol and membrane proteins is that effects evolve faster, and that cholesterol is truly removed from the membrane rather than converted, with the generation of enzymatic products being inherently at risk for giving rise to side effects. Moreover, MβCD acts strictly at the membrane surface [53]. We added 10 mM MβCD to the extracellular solution in the beginning of whole-cell patch clamp measurements on TRPV6 WT-transfected HEK293 cells and continued to record Ca^2+^ influx currents for 20 min. Control experiments were exerted analogously yet in the absence of MβCD. Again, statistical evaluation showed that application of 10 mM MβCD neither changed maximum TRPV6 currents nor the SCDI behavior of the channel in a significant manner (Figure 4A–C). In a second approach, we preincubated analogously transfected cells for 15 min at 37 °C in 0 mM Ca^2+^ solution with 10 mM MβCD. As a control, cells were preincubated for 15 min at 37 °C in 0 mM Ca^2+^ solution without adding MβCD. In agreement with what was observed for acute application, preincubation with 10 mM MβCD also revealed no significant difference regarding maximum currents and SCDI in comparison to the control experiments (Figure 4D–F). Although statistical significance was not reached in both cases, mean currents of those cells exposed to MβCD appeared to nevertheless be somewhat higher compared to the untreated control, while the averaged extent of SCDI was reduced in comparison with the untreated control.

### 3.6. Cholesterol Sequestration at Different Stages of Protein Expression Using Filipin Does Not Significantly Alter Maximum Currents and SCDI of TRPV6 WT

**Figure 5:**
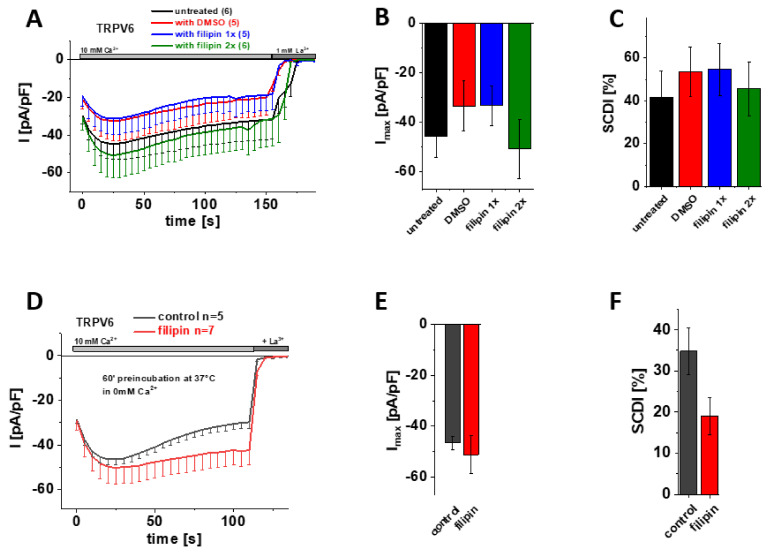
**Treatment with filipin has no effect on maximum currents and SCDI of TRPV6 WT currents.** (**A**) Averaged time course of whole-cell currents (mean ± SEM) recorded from HEK293 cells expressing TRPV6 WT. Cells were treated with DMSO (control, red), with 1 µg/µL filipin upon transfection (blue, filipin 1x) or with 1 µg/µL filipin upon transfection and media exchange (green, filipin 2x). Untreated cells (black) were taken as an additional control. Measurements were carried out in 10 mM Ca^2+^ extracellular solution and currents were blocked by 1 mM La^3+^ at the end of the experiments, as indicated by the grey bars. (**B**) Block diagram of maximum currents according to measurements in (**A**). (**C**) Block diagram of the extent of SCDI in % according to measurements in (**A**). (**D**) Averaged time course of whole-cell currents (mean ± SEM) recorded from HEK293 cells expressing TRPV6 WT. Cells were preincubated in 0 mM Ca^2+^ extracellular solution with (red) and without (control, black) 1 µg/µL filipin at 37 °C for 60 minutes. Measurements were carried out in 10 mM Ca^2+^ extracellular solution and currents were blocked by 1 mM La^3+^ at the end of the experiments, as indicated by the grey bars. (**E**) Block diagram of maximum currents according to measurements in (**D**). (**F**) Block diagram of the extent of SCDI in % according to measurements in (**D**).

Filipin, which is actually a mixture of polyene antibiotics, engages complexes with cholesterol, thereby interfering with protein–cholesterol interactions and distorting the integrity of sterol-containing membranes [54,55]. Although filipin is frequently used to label cholesterol within membranes as well, we again took advantage of this substance for manipulating presumptive cholesterol-TRPV6 interactions. Therefore, we added 1 µg/µL filipin already upon transfection of the cells (Figure 5A–C; blue) or additionally upon media exchange (Figure 5A–C; green) so that the agent was present for about 4 h after transfection or throughout all stages of protein expression, respectively. As filipin is dissolved in DMSO, we also added an equivalent amount of DMSO to the transfection mix for the control measurements. Transiently transfected HEK293 cells lacking any filipin or DMSO treatment (Figure 5A–C; untreated) served as an additional control. Consistent with the cholesterol depletion experiments shown above, patch clamp recordings in the whole-cell mode revealed no significant difference regarding maximum current or SCDI of TRPV6 WT between cells treated with either filipin or DMSO or untreated control cells (Figure 5A–C).

Finally, we also tested the experimental approach of preincubation with filipin. Therefore, we incubated transiently transfected HEK293 cells for 60 min at 37 °C in 0 mM Ca^2+^ extracellular solution with and without supplementing 1µg/µL filipin, respectively. Again, the subsequent patch clamp measurements revealed no significant difference in maximum TRPV6 WT currents upon cholesterol depletion via filipin (Figure 5 D–F).

Together, none of the approaches to interfere with protein–cholesterol interactions by means of filipin treatment showed a significant change in maximum current or SCDI of TRPV6 WT currents, agreeing with the afore presented data on cholesterol depletion by MβCD or enzymatic treatment (CO).

### 3.7. Preincubation with Cholesterol Oxidase Has No Effect on TRPV6 WT Inhibition by Cis-22a

**Figure 6:**
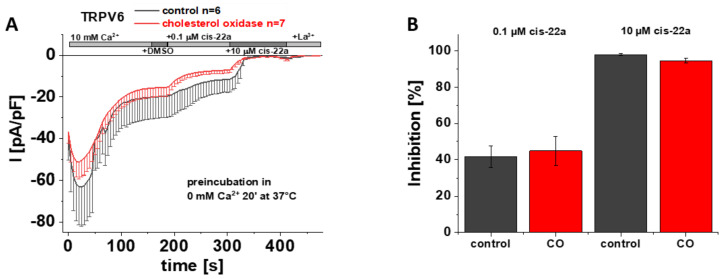
**Preincubation with cholesterol oxidase has no effect on TRPV6 WT inhibition by cis-22a.** (**A**) Averaged time course of whole-cell currents (mean ± SEM) recorded from HEK293 cells expressing TRPV6 WT. Cells were preincubated in 0 mM Ca^2+^ extracellular solution with (red) and without (control, black) 2 U/mL CO at 37 °C for 20 minutes. Throughout the experiment, application of 10 mM Ca^2+^ was followed by consecutive addition of DMSO, 0.1 µM cis-22a, 10 µM cis-22a and 1 mM La^3+^, as indicated by the grey bars. (**B**) Block diagram of the extent of inhibition in % by 0.1 µM (left) and 10 µM (right) cis-22a according to the measurements in (**A**). There is no statistical significance (*p* < 0.05) in comparison with TRPV6 WT.

As previously mentioned, LBS-2 represents a potential binding site for cis-22a and as presented earlier herein, mutations of key residues thereof lead to a significantly reduced ability of this small molecule blocker to inhibit TRPV6 currents (Figure 2A,D). This led us to speculate whether cholesterol was too tightly bound within the channel to get effectively depleted and if the concurrent action of cholesterol depletion and exposure to cis-22a might alter the extent of inhibition of TRPV6 currents by cis-22a. If intending to investigate blockage by cis-22a, awareness is needed that individual measurements have to continue through a rather long period of time. This is on the one hand explained by the need to allow SCDI to reach a steady-state and on the other by the time needed to establish a current plateau after each application step, throughout which the membrane/seal must not rupture. Due to the mentioned requirements, we chose CO to reduce the amount of cholesterol in the membrane for these specific experiments because it represents the mildest among the mentioned cholesterol depletion efforts. The latter is explained by cholesterol getting neither directly extracted from the membrane upon CO treatment nor aggregated as in the case of the other two approaches, respectively [48]. We preincubated transiently transfected HEK293 cells for 20 minutes at 37 °C in 0 mM Ca^2+^ extracellular solution with or without 2 U/mL CO, in analogy to electrophysiological recordings shown in Figure 3 and Figure S2. Implementing the same measurement protocol for the recordings as shown in Figure 2A, it turned out that the response of TRPV6 WT to cis-22a is indifferent to whether or not CO was present during the preliminary incubation. Indeed, the inhibitory effect of cis-22a was neither changed for the low (0.1 µM) nor for the high (10 µM) concentration of the blocker, respectively (Figure 6A,B).

## 4. Discussion

Lipids have gained a high reputation as endogenous modulators of ion channels and as signaling molecules rather than being exclusively perceived as construction elements of membranes. Within the cryo-EM structure of open human TRPV6 channels, four types of presumptive lipid binding sites have been identified (LBS-1–LBS-4) [32]. In the present study, we found that single point mutations altering charges in LBS-2 or polar residues (Q483K, K484A, G488R and Q596E) reduce maximum currents and slow Ca^2+^-dependent inactivation (SCDI) of the mutant channels compared to TRPV6 WT. LBS-2 represents, apart from the pore, a binding site for the small molecule inhibitor cis-22a [30]. Indeed, in electrophysiological recordings, we found that any of the four mutants is significantly less inhibited by 0.1 μM cis-22a, a dosage approximately matching the IC_50_ value determined for the wild-type [30]. TRPV6 K484A and G488R also displayed a significant reduction in the inhibition by 10 μM cis-22a in Fura-2 as well as patch-clamp measurements, as did Q596E if investigated with the latter technique. Altogether, TRPV6 G488R was, among all analyzed mutants, most resistant to the specific inhibitor and also showed the lowest level of SCID. In contrast to LBS-2 mutagenesis, none of our treatments with CO, MβCD or filipin, in order to interfere with interactions between cholesterol and membrane proteins, resulted in significant alterations in maximum currents and inactivation of TRPV6. Nevertheless, it needs to be mentioned that mean currents of MβCD treated cells (Figure 4) and SCDI of cells recorded after preincubation with filipin before the measurement (Figure 5D,E) slightly deviated from the respective controls, although not significantly.

The introduction of both, positive and negative charges as well as neutralization of basic residues within LBS-2 to inversely affect lipid binding had the same effect on the characteristics of the channel. Lipid engagement at this site is assumed to be activating, given that the TRPV6 R470E mutant, which was reported to show a considerably slower Ca^2+^ uptake compared to TRPV6 WT and is thus considered as a representative of the closed state, shows a smaller size of LBS-2. Therefore, one might expect that the strengthening of lipid binding promotes channel opening and/or reduces channel inactivation. Vice versa, it would be reasonable to assume that interfering with lipid binding abilities reduces channel opening and eventually promotes SCDI. Yet, it needs to be considered that the lipid-bound states of the mutants are likely rather different. Those with an eventually reduced affinity on the level of single channels, may have inherently a lower amount of lipid captured, and/or with respect to individual cells, the number of channels with a saturated LBS-2 might be lower. An eventually lower number of dissociable lipids compared to the wild-type state might explain the compromised inactivation, presuming that loss of the lipid is responsible for inactivation. Instead, reductions in inactivation seen with an additional positive charge in LBS-2 might indeed represent the consequence of a firmer association which hinders loss of the ligand, as expected. Although, at the current stage, we can also not exclude a steric effect of the herein investigated mutants. Glycine, for instance, holds an outstanding role in protein architecture, as it allows for structural flexibility rather than rigidity. Thereby, replacement of G488 with a sterically and chemically very different arginine residue within a key region of TRPV6 might have had consequences on the channel apart from lipid binding itself, such as relaying the signal of LBS-2 engagement to the pore. Similarly, the introduced acidic residue instead of the polar glutamine in the case of the Q596E mutant might have allowed for the formation of salt bridges with appropriately positioned basic residues of LBS-2, thereby interfering with the natural functioning of the channel. Still, at least for TRPV6 G488R, there are different lines of evidence for a change in the lipid binding affinity [36].

Considering the cryo-EM structure of the open TRPV6 resolved by McGoldrick et al. in 2018, the LBS-2 density likely represents cholesterol or cholesterol hemi succinate (CHS), given that the latter was highly abundant upon protein purification in the study concerned [32]. Indeed, sequence analysis also predicts several cholesterol recognition amino acid consensus (CRAC) motifs (K/R)-X_1-5_-(Y/F)-X_1-5_-(L/V) and CARC motifs (K/R)-X_1-5_-(Y/F)-X_1-5_-(L/V) to be present throughout TRPV6, also involving LBS-2 residues [56,57,58]. Yet, our present results indicate that, at least upon overexpression of TRPV6 in HEK293 cells, cholesterol plays a minor role in TRPV6 regulation. In contrast, Kever et al. reported that treatment of Jurkat T lymphocytes with MβCD reduces TRPV6 activity, evoked by a reduction in cell surface expression of endogenous TRPV6 upon cholesterol depletion [33,34]. Interestingly, internalization of other proteins was found to be inhibited by filipin or MβCD in a concentration-dependent manner, as it was reported for the lymphotoxin β receptor and, among others, for the serotonin_1A_ receptor [59,60,61]. However, the seemingly contradictory results of a reduced TRPV6 activity after exposing Jurkat T cells to MβCD while we observed the reverse, yet statistically non-significant effect on maximum currents upon expressing the channel in HEK293 cells, might indicate some cell-type specificity in TRPV6 regulation by cholesterol. Thereby, differences in the function of both cell types need to be considered, as the MβCD application was found to affect cell adhesion and migration of Jurkat T lymphocytes [34], while HEK293 cells are inherently adherent. The former was supposed to relate to an association of TRPV6 with Lck kinase within lipid rafts, which is prevented upon the distortion of lipid rafts due to cholesterol depletion [34]. Moreover, awareness is needed that the regulatory effects of endogenous modulators might be attenuated in settings implementing overexpression of the target protein [62]. Additionally, it needs to be emphasized that translation of endogenous TRPV6 proteins starts at an ACG codon which is erroneously decoded to methionine, while the variant that is frequently used for overexpression which was also implemented in the present study starts at the first AUG codon, thus lacking the N-terminal most in 40 amino acids [63]. Thereby, it might be speculated that the concerning stretch is involved in cholesterol regulation, eventually in an indirect manner, e.g., by containing interaction sites for accessory proteins. In the case of Orai1, for instance, interactions with Caveolin 1 (Cav1) seem to promote accumulation of the former within subregions of a high cholesterol content and a specific architecture that is referred to as caveolae and thereby to foster regulation by cholesterol [64,65]. Interestingly, for CRAC channels, the effects of cholesterol depletion are apparently dependent on the activation state, promoting calcium entry if the channel is already active, while removal of this specific lipid before STIM1-Orai1 complexes are formed has the reverse effect [51,66,67].

While structurally identified as a binding site, the role of the association of cis-22a with LBS-2 is rather elusive. In contrast, resolving the structure of TRPV6 in complex with econazole allowed for the delineation of a conclusive mechanism of channel inhibition by lipid replacement. The substance binds at the interface of S5 and S4 of two adjacent subunits, with hydrophobic residues lining the binding site. The association with econazole instead of the else bound, larger lipid was reported to gives rise to a void, resulting in the residues Q473 and R589 being further apart. This larger distance precludes the formation of a salt bridge between them as observed in the open state, otherwise compensating for the energetic expense of the α-to-π helical transition and thus resulting in channel closure [23]. Similar to the size difference reported for econazole, the LBS-2 density is decreased when cis-22a is present. Yet, elaborating a mechanism of how cis-22a binding to LBS-2 affects the channel is considerably intricate, given the presence of two different binding sites. Furthermore, the pore site seems to inherently own a higher relevance for channel inhibition. Yet, the hypothesis was raised that mutations within LBS-2 allosterically alter the pore binding site, since concurrent mutation of both sites, as in the case of the TRPV6 R470A W583F double mutant, has been found to reduce the inhibitory potential of cis-22a much more than the single point mutations individually and to an extent, stronger than the additive [30].

Nonetheless, considering the pore as the main binding site, it is even more surprising that TRPV6 G488R was essentially resistant to inhibition by 10 μM cis-22a. Additionally, the SCDI of this mutant was strongly compromised in our experiments as well as in previous measurements of Velisetty and colleagues, even upon implementing a different strategy to monitor inactivation. In the same study, a considerably higher affinity of the mutant for PIP_2_ was quantified in dose–response measurements, which is, among others, further supported by a lower sensitivity of the mutant to the depletion of this lipid compared to TRPV6 WT [36].

## 5. Conclusions

Collectively, our non-significant effects of cholesterol depletion but rather strong consequences of site-directed mutagenesis within LBS-2 would favor that PIP_2_, the alternative lipid matching the chemical binding prerequisites, predominates TRPV6 regulation. Although we cannot exclude that overexpression obscured, at least in parts, a modulatory role of cholesterol on TRPV6, this notion is, apart from data on G488R, supported by molecular dynamic (MD) simulations predicting K484, in addition to R302 and R305, as PIP_2_ interacting residues [47]. Furthermore, a reduced affinity of TRPV6 K484Q for PIP_2_ has been identified in biochemical experiments [37]. Thereby, the poor fit of PIP_2_ with the LBS-2 density might be due to the replacement of the endogenously bound lipid by abundant CHS, an artifact also known from other channels [47,68].

## Data Availability

This study includes no data deposited in external repositories. Original data related to this paper will be provided upon reasonable request by the corresponding author.

## Supplementary Materials

The following supporting information can be downloaded at: https://www.mdpi.com/article/10.3390/biom12060804/s1, Figure S1: Ratiometric Fura-2 measurements on TRPV6 WT and mutants. Figure S2: Preincubation with cholesterol oxidase significantly increases maximum CRAC currents.
