# Supplementary material for: TRPV6 Regulation by Cis-22a and Cholesterol
Source: Biomolecules. 2022 Jun 8;12(6):804. doi: 10.3390/biom12060804 (PMC9221249; doi:10.3390/biom12060804)
Supplement: Supplementary file 1 [file biomolecules-12-00804-s001.zip › biomolecules-1760756-supplementary.pdf]

Figure S1

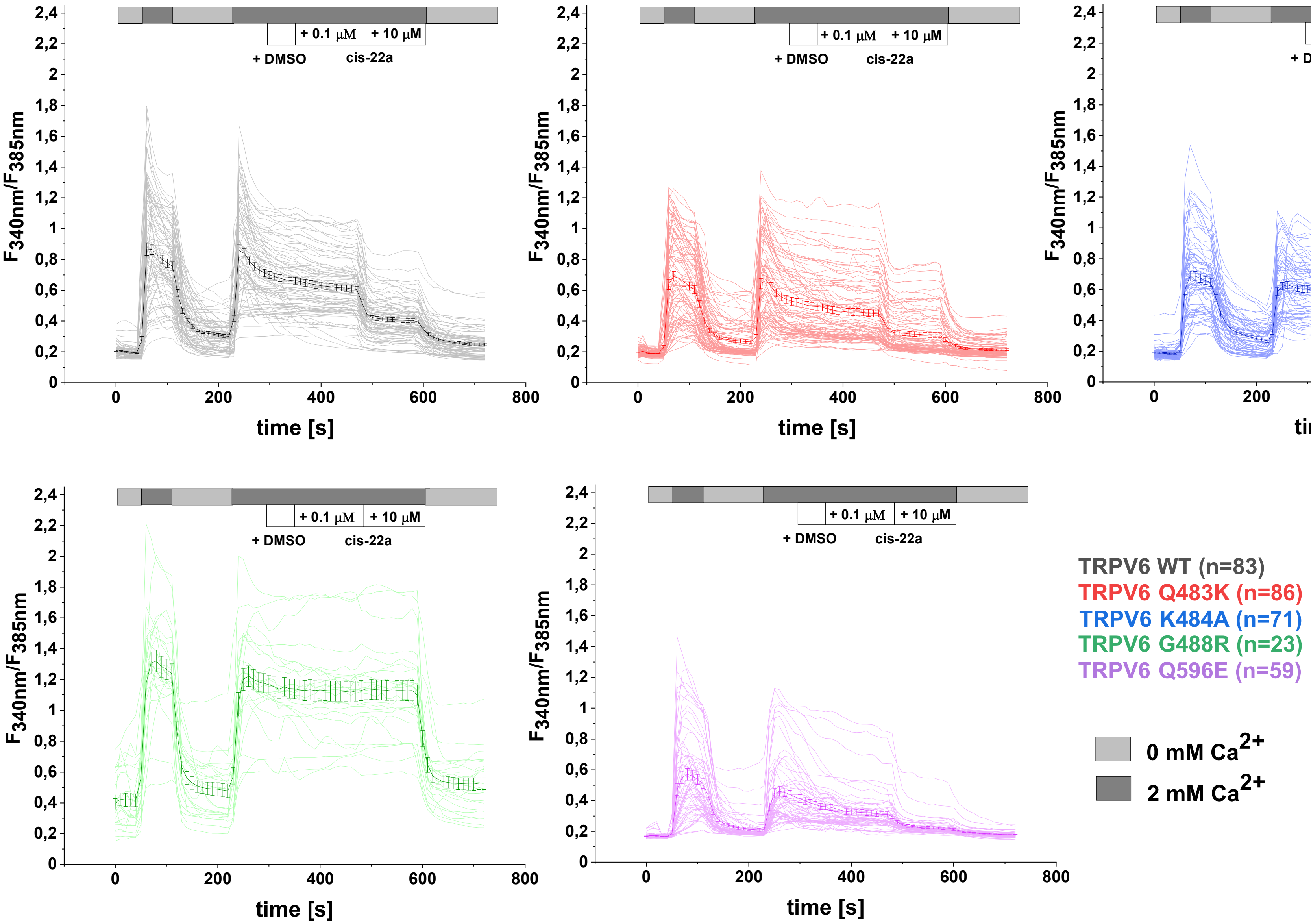

Figure S1: Ratiometric Fura-2 measurements on TRPV6 WT and mutants. Changes in the intracellular Ca^{2+} level of HEK293 cells overexpressing human TRPV6 WT or the respective mutant upon perfusion with Ca^{2+}-free ECS, 2 mM Ca^{2+}-containing ECS, 0.1 vol%DMSO, 0.1 µM and 10 µM cis-22a in 2 mM Ca^{2+}-containing ECS, as indicated by the bars. Fura-2 emission was monitored in intervals of 10 s upon alternating excitation at 340 nm and 385 nm. After background subtraction, the ratio of the respective emission intensities was determined (F_{340nm}/ F_{385nm}). The respective mean values (± SEM) of 23-86 cells, measured on 2-4 days, are overlayed on the individual traces.

Figure S2

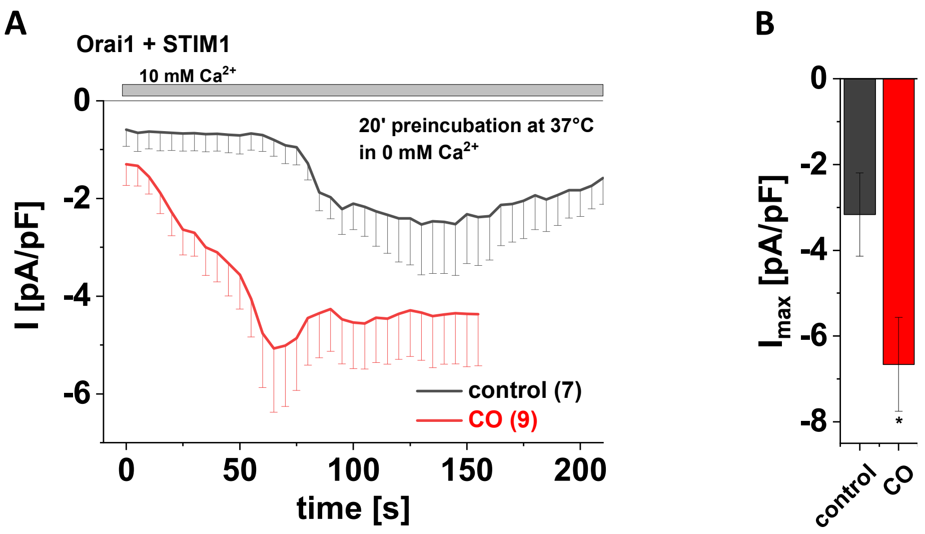

Figure S2: Preincubation with cholesterol oxidase significantly increases maximum CRAC currents. (A) Averaged time course of whole-cell currents (mean ± SEM) recorded from HEK 293 cells coexpressing Orai1 and STIM1. Cells were preincubated in 0 mM Ca_{2+} extracellular solution with (red) and without (control, black) 2 U/ml CO at 37 °C for 20 minutes. Measurements were carried out in 10 mM Ca_{2+} extracellular solution. **(B)** Block diagram of maximum currents according to measurements in (A). The asterisk (*) highlights the statistical significance ($p < 0.05$) in comparison to control experiments.
